# Sexual function and fertility in young female adults surgically treated for anorectal malformations

**DOI:** 10.1007/s00383-024-05847-1

**Published:** 2024-10-10

**Authors:** Joshua Gertler, Jenny Oddsberg, Anna Gunnarsdóttir, Anna Svenningsson, Tomas Wester, Lisa Örtqvist

**Affiliations:** 1 Unit of Pediatric Surgery, Astrid Lindgren’s Children’s Hospital, Karolinska University Hospital, Eugeniavägen C11:33 Solna, 17176 Stockholm, Sweden; 2 Department of Women’s and Children’s Health, Karolinska Institutet, Stockholm, Sweden

**Keywords:** Anorectal malformation, Female sexual function, QoL, Bowel function, Urinary tract function, Surgery

## Abstract

**Purpose:**

The aim was to investigate sexual function and fertility in female adults operated on for anorectal malformations (ARM).

**Methods:**

This was a cross-sectional questionnaire-based study including female adult patients treated for ARM at our institution between 1994 and 2003. Sexual function in females was assessed using the Profile of Sexual Function (PFSF). Additional questions regarding fertility were answered by the participants. Patient characteristics were retrospectively retrieved from the medical records and descriptive statistics were used for analysis. Sexual function outcomes were compared to a control group from a previously published group of females. Composite outcome analysis was performed using previously published data to determine the potential impact of bowel function and health-related quality of life on sexual function. The ethics review authorities approved the study.

**Results:**

A total of 14 of 30 (46.7%) females responded to the questionnaires and had a mean age of 21.1 years (range 18–26). No association was found between PFSF and age or bowel function (Bowel Function Score), however, a strong correlation was found between PFSF and health-related quality of life (HRQoL) with a Spearman correlation of *ρ* 0.82 (*p* = 0.0011). The general satisfaction question was strongly associated with their total PFSF score (*ρ* = 0.71, *p* = 0.0092). Except for the “desire” item, the females in this cohort did not have significantly worse sexual function than the control population(*p* = 0.015). Ten of fourteen (71.4%) females had had their sexual debut at a mean age of 16.3 years and two of these women (20%) have been pregnant. All females had had menarche at a mean age of 12.7 years.

**Conclusion:**

Sexual function in adult females was comparable to healthy controls except for the “desire” item where the cohort reported poorer outcomes. The cohort’s sexual function had a direct association with their reported HRQoL where individuals with worse HRQoL also reported poorer sexual function.

**Level of evidence:**

III

**Supplementary Information:**

The online version contains supplementary material available at 10.1007/s00383-024-05847-1.

## Introduction

Congenital anorectal malformations (ARM) are a spectrum of congenital anomalies requiring corrective surgery for survival. The birth prevalence of ARM in Sweden is approximately 1:3000 [1, 2]. A large registry-based study of 17 European regions showed that associated malformations occurred in 50–67% of patients with ARM [3]. These associated malformations potentially have a further impact on the functional and psychological outcomes of patients with ARM.

Peña and DeVries laid the foundations of the currently used surgical technique, posterior sagittal ano-recto-plasty (PSARP), in the early 1980’s[4]. From that era, survival rates in infants with ARMs have improved alluding to the progress of surgical and neonatal care. Consequently, a shift in treatment goals followed from survival to optimizing functional and psychological outcomes. Our research group has recently reported on bowel function, urinary tract function and health-related quality of life (HRQoL) into adulthood for male and female patients[5, 6]. A significant number of adult patients are known to have persistent troubles with functional and sexual outcomes impacting their quality of life [7]. It is also established that sexual dysfunction was common in ARM patients operated on in the pre-PSARP era [8, 9] and a later coital debut has been reported compared to healthy controls [10, 11]. Further investigation of composite and controlled data is deemed imperative [6, 11]. In a recent editorial, it was stated that “Developing…strategies for long-term follow up…of pediatric surgical patients who may have altered sexual function and fertility should be a priority for our field” [12]. The aim of this study was to investigate female patients’ sexual function and fertility in the post-PSARP era.

## Methods

### Study design

This was cross-sectional questionnaire-based study. The study was registered in ClinicalTrials.gov (NCT04901819).

### Study setting

Individuals with ARM managed at the Unit of Pediatric Surgery at Karolinska University Hospital, Stockholm, Sweden. In 2024, Sweden had a population of roughly 10.6 million persons.

### Participants

All surgically managed females with ARM at our institution between 1994 and 2003 were eligible for the study. A database of identified eligible patients was created. The included ARM subtypes were perineal fistulas, vestibular fistulas, recto-vaginal fistulas as well as atresias without fistulas. Deceased patients, patients with cloacal malformations and patients without surgical interventions were excluded from the study. Furthermore, patients with Currarino syndrome, Down’s syndrome and patients with major intellectual disabilities were excluded from the study. After informed consent, participants were asked to answer a composite questionnaire pertaining to the focus of the study. Participants had the option to respond using paper mail or a digital platform (REDCap). A reminder was mailed to non-respondents after 4 and 8 weeks, respectively.

Results of a previously published control group of 171 healthy age-matched individuals were used for analysis regarding female sexual function [13]. The individuals in the control group had been randomized in a double-blinded study, received placebo oral contraceptive and were asked to answer a sexual function questionnaire. The same questionnaire, Profile of Female Sexual Function (PFSF), was employed in our study.

### Data sources and variables

#### Patient characteristics

Patient characteristics and clinical details were recorded retrospectively from the medical records. These data included information about associated anomalies, ARM subtype according to Krickenbeck Classification [14], surgical procedures and age at the time of the study. The follow-up date was set for the 15th of June 2021.

#### Fertility

General questions were asked about, menarche, dysmenorrhea, coital debut, pregnancies, deliveries, the impact of their ARM on their sexual function and civil status. Fertility was defined as the rate of childbirth.

#### Sexual function

Sexual function for our cohort was examined using the validated PFSF questionnaire [13, 15], where our primary outcome consisted of the average normalized score of the seven domains referred to as ‘PFSF total score’ ranging from 0 to 100. The normalized scores of individual domains were examined as secondary outcomes. The PFSF instrument contains 37 items in seven domains (sexual desire, arousal, orgasm, sexual pleasure, sexual concerns, sexual responsiveness, and sexual self-image) and a single-item measure of overall ‘sexuality satisfaction’. A score < 40 in the sexual desire domain has been previously described as “low sexual desire” [16, 17].

#### Bowel function

Bowel function was used as a composite outcome to assess sexual function using previously published data pertaining to the same individuals as the current cohort [5]. A bowel function score (BFS) of ≥ 17 of a maximum of 20 was used as an indicator of well-preserved bowel function as described previously by Kyrklund et al. [18]. Bowel function was evaluated in patients regardless of the use of laxatives, enemas, or antidiarrheal medication.

#### health-related quality of life (HRQoL)

Likewise, previously published data on HRQoL pertaining to this cohort was used to assess its potential impact on sexual function[5]. The same cohort of adults (18 to 26 years old) answered a validated instrument Psychological General Well-Being Index (PGWBI) [19–21]. The instrument includes six dimensions comprising a total of 22 items with a maximal total score of 110, the higher the score the better HRQoL. The dimensions include Anxiety, Depressed Mood, Positive Well-being, Self-Control, General Health, and Vitality. Outcomes of the survey are interpreted as follows; 0–60 “Severe Distress”, 61–71 “Moderate Distress”, 72–92 “No Distress”, and 93–110 “Positive Well-being” [21].

## Statistical methods

Categorical variables were presented using frequencies and proportions whereas continuous variables were presented as mean with standard deviation (SD). PFSF values were compared between patients and age-matched female reference values using independent t-tests. The Wilcoxon Sum Rank Test was used to compare the PFSF total score between categorical values of BFS and HRQoL. A significance level of p < 0.05 was used.

The Spearman correlation test was used to analyze the association between PFSF and several variables (age, BFS, overall sexual satisfaction, HRQoL) where rho(ρ) > 0.7 = strong correlation, > 0.4 moderate association, 0.2–0.4 = no correlation.

### Ethical considerations

The study was approved by the Swedish Ethical Review Authorities.

## Results

### Patient characteristics

**Fig. 1:**
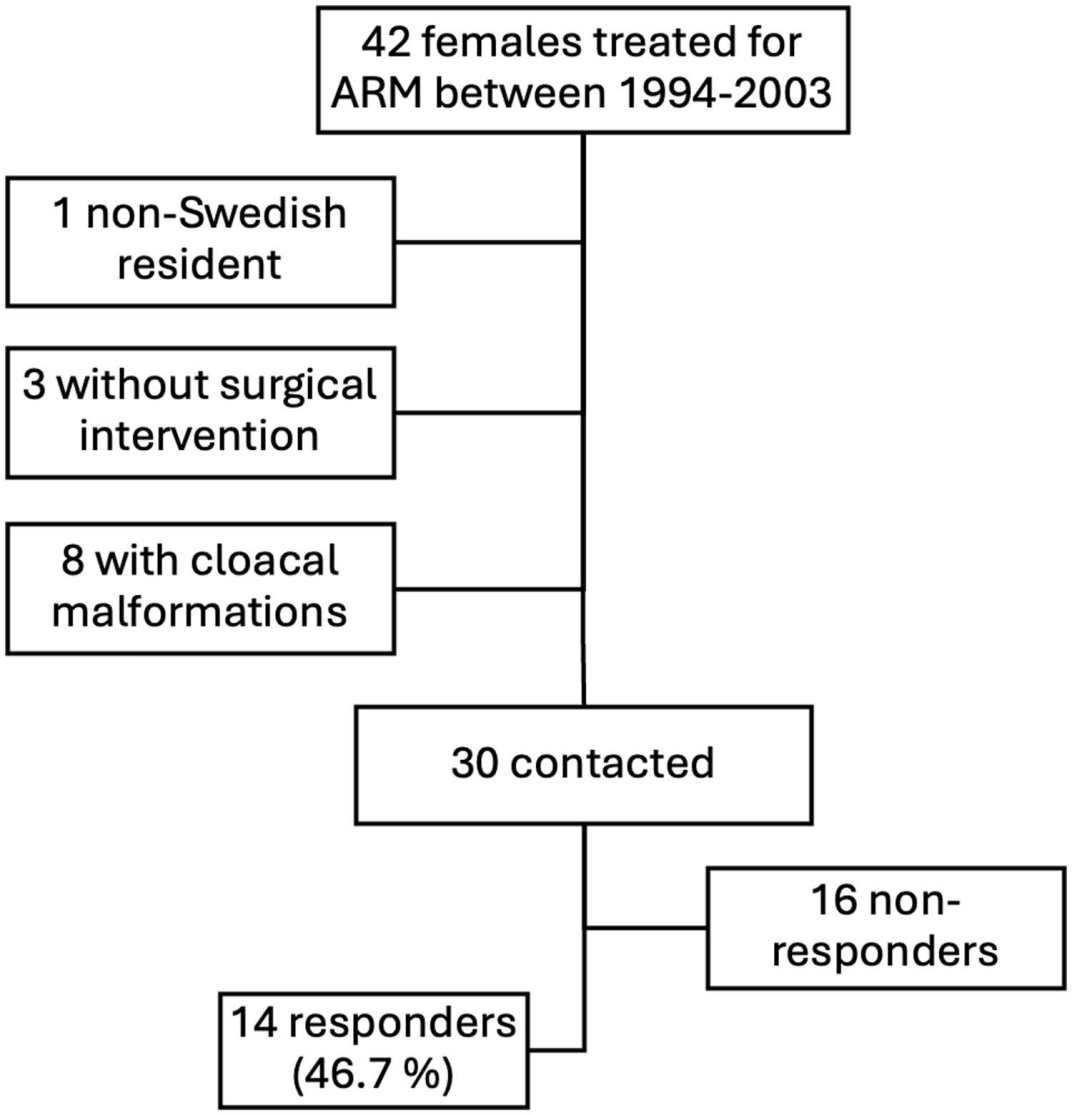
The participant’s inclusion process

The inclusion process is summarized in Fig. 1

All included participants responded to the questions concerning menarche, sexual activity and fertility at a mean age of 21.1 years (SD = 2.7). Twelve of these also responded to the PFSF questionnaire at a mean age of 21.4 (SD = 2.7) whilst the control group had a mean age of 24.1 (SD = 4), p = 0.007. Patient characteristics are summarized in Table 1. Half of the participants had been treated for a vestibular fistula and nearly 30% for a perineal fistula. Missing data was high pertaining to associated malformation, yet 71.4% of participants with data had an associated malformation. No patients had permanent stomas or a urinary diversion at the time of the study.

**Table 1:** Patient Characteristics of the 14 responders

	*N* (%)	Missing data (*n*)
Mean age at follow-up, Years (SD)	21.1 (2.7 )	0
Type of ARM		0
Perineal Fistula	4 (28.6)	
Vestibular Fistula	7 (50)	
Atresia without Fistula	1 (7.1)	
Recto-vaginal Fistula	2 (14.3)	
Associated malformations	5 (35.7)	7
Esophageal atresia	1 (7.1)	
Cardiac malformation	1 (7.1)	
Urinary tract anomalies	1 (7.1)	
Vertebral anomalies	1 (7.1)	
Tracheal anomalies	0	
Limb abnormalities	1 (7.1)	
VACTERL association	0	
Spinal cord abnormality	1 (7.1)	
Current Occupation among patients ≥ 18 years, n = 12		
Student	6 (42.9)	
Full-time employed	5 (35.8)	
Part-time employed	4 (28.6)	
On sick-leave	0	

IQR Interquartile range; *ARM* anorectal malformation; *ACE* antegrade continence enema; *VACTERL* vertebral-anal-cardiac-tracheo-esophageal-renal-limb)

### Gynecological, sexual and obstetrical outcomes

The responses are summarized in Table 2. All females had had menarche at a mean age of 12.7 (SD = 0.91) years. Out of the 14 females, ten (71.4%) had had their sexual debut at a mean age of 16.3 (SD = 1.57) years and two of these women (20%) had been or was currently pregnant. The patient who had delivered with a caesarian section was recommended this delivery mode due to her medical history of ARM. The second female had a caesarian section planned at the time of the study.

**Table 2:** Fertility and sexuality characteristics within the cohort, *n* = 14

	*N* (%)	Commentary
Presence of menstruation	12 (85.7)	2 without had hormonal IUD
Mean age at Menarche, years (SD)	12.7 (0.91)	
Regular menstruation	9 (64.3)	
Issues with menstruation	1 (7.1)	Caused by ovarial cysts
Use of medicinal contraception	4 (28.6)	
Ever been pregnant?	2 (14.3)	3 and 1 time(s), respectively
Age of pregnancies, mean years	18.75	
Deliveries	1	1 other patient currently pregnant
Mode of delivery	C-section	1 other patient planned for C-Section
If not previously pregnant, have you tried to become pregnant	0	
Marital status		
Married	2 (14.3)	
Partner but not married	3 (21.4)	
No partner	9 (64.3)	
Coital debut	10 (71.4)	
Age of coital debut, mean (range)	16.3 (15–20)	
Of those with coital debut, can you have penetrating sex?	10 (100)	
To what extent does your ARM impact your sexual life (*n* = 10)		Free text comments
No extent	3 (30)	“I don’t know what my ARM impacts”
Low extent	4 (40)	“Feel ashamed, incontinence issues”
Medium extent	3 (30)	“Ashamed of my appearance”
High extent	0	

*IUD* Intrauterine device; *ARM* anorectal malformation

### Profile of female sexual function

**Fig. 2:**
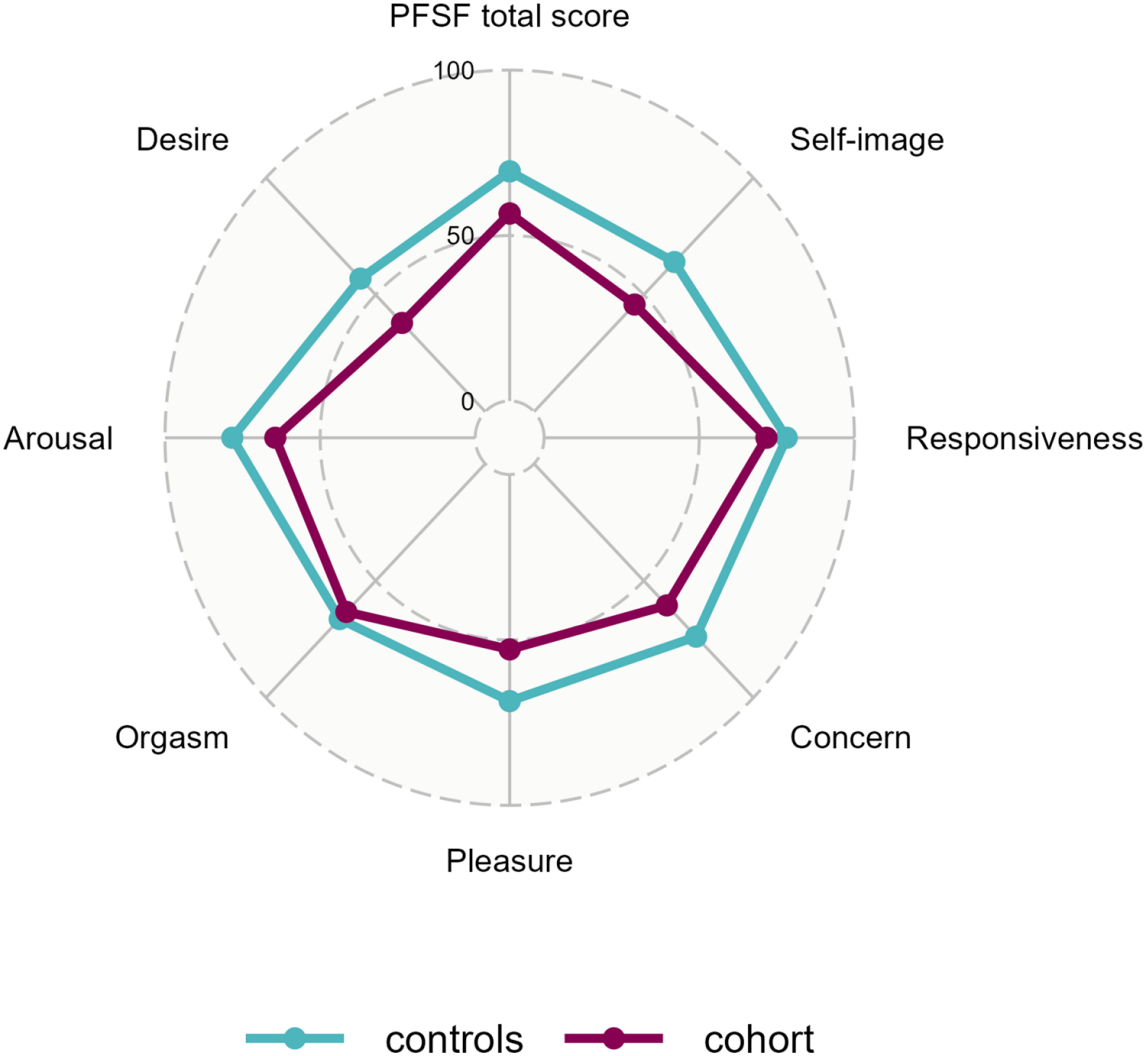
Radial diagram of PFSF cohort and controls mean values per domain. (Profile of Female Sexual Function, *PFSF*)

Except for the “desire” item (*p* = 0.015), the females in our cohort did not have significantly worse sexual function than the control population, visually depicted in Fig. 2. However, the cohort did score worse than the controls across all domains. The numerical data is summarized in Supplementary Table 1.

### Composite outcomes

**Fig. 3:**
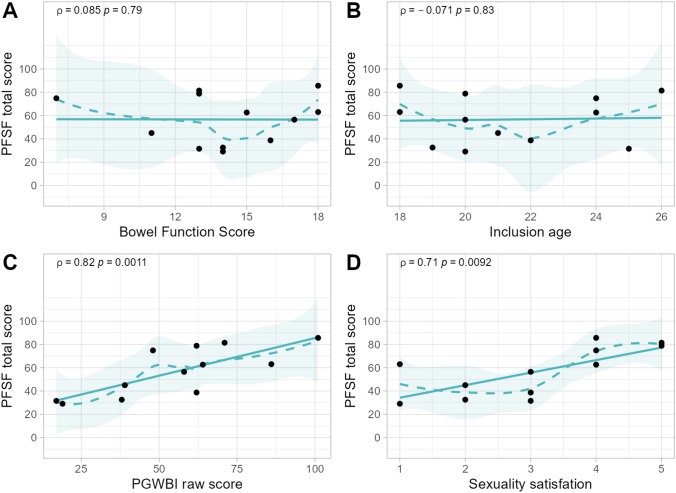
Graphs showing the degree of correlation of PFSF with (**A**) BFS, (**B**) inclusion age, (**C**) HR-QoL and (**D**) satisfaction of sexuality, respectively. Spearman correlation where rho (ρ) > 0.7 = strong correlation. Significance set as *p* < 0.05. (Abbreviations: PFSF – Profile of Female Sexual Function, BFS – Bowel Function Score, PGWBI – Psychological General Well-Being Index, HR-QoL – Health-Related Quality of Life)

Spearman correlation test (Rho = ρ) was performed to evaluate PFSF correlation to age, BFS, HRQoL and the general sexuality satisfaction question. No association was found to age or BFS, however, a strong correlation was found between PFSF and health-related quality of life (HRQoL) with a Spearman correlation of *ρ* 0.82 (*p* = 0.0011)(Fig. 3C). The general satisfaction question was strongly associated to their total PFSF score (*ρ* = 0.71, *p* = 0.0092)(Fig. 3D).

**Fig. 4:**
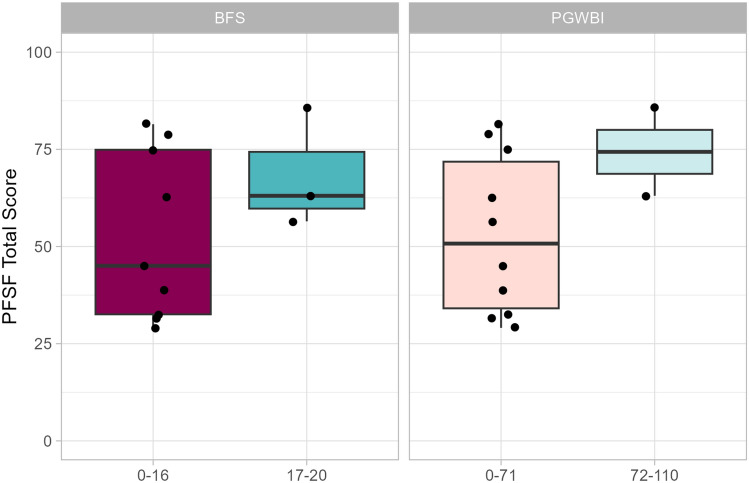
Boxplots depicting PFSF total scores related to brackets of BFS and PGWBI. (Profile of Female Sexual Function, *PFSF*; Bowel Function Score, *BFS*; Psychological General Well-Being Index, *PGWBI*)

Using the Wilcoxon Sum Rank test, it was also investigated if specific brackets within BFS and HRQoL were associated with PFSF outcome. As previously described, a BFS of ≥ 17 of a maximum of 20 was used as an indicator of well-preserved bowel function and a PGWBI raw score > 72/110 relates to individuals not having psychological distress. Although not statistically significant (*p* = 0.282 and 0.182, respectively), a visual trend could be seen in Fig. 4 where the positive brackets of BFS and PGWBI yield higher PFSF values.

## Discussion

### Key findings

In this cohort of young adult females operated on for ARM, menarche occurred at a mean age of 12.7 years, most of them had had coital debut at a mean age of 16.3 years. The cohort scored lower in all domains of the PFSF when compared to controls and significantly lower in the “desire” domain. A strong correlation was shown between HRQoL and PFSF as well as between overall sexuality satisfaction and PFSF.

### Interpretation

Research in this field to this date is still scanty. A systematic review in 2021 found 4 articles describing long-term sexual function in females and concluded that female 50% (95% CI 34–66%) of females had sexual dysfunction [22]. Due to the lack of studies and in general small study populations, it is difficult to draw conclusions, and it should be noted that sexual dysfunction in this systematic review was measured in different ways, where both qualitative and questionnaire-based (FSFI -Female Sexual Function Index) measures were used. Further, this limits the possibility to stratify by ARM subtype which clearly is of the essence for ARM patients. One study included 46 patients regarding male sexual function stratified results by ARM subtype, yet no statistical differences were found regarding the prevalence of sexual dysfunction [23].

Pertaining to anatomical and phenotypical differences in ARM, gender-specific research has yielded major differences between the two sexes[5, 6]. Differences in measurement tools and questionnaires for sexual function also suggest that subgroups should be analyzed separately. We separately analyzed a subset of male patients, however, only 5 of the 13 males included provided sufficient information but had had coital debut. Of interest, those who did complete the IIEF-5 questionnaire, all scored between 24 and 25 points out of a possible 25, thereby scoring in the “no erectile dysfunction” bracket (data not published).

The choice of a suitable questionnaire was crucial for this study. Having healthy controls in a similar age span to compare our cohort to was of the essence and this was the major reason for choosing the PFSF, which is also a validated questionnaire [15]. Another well-used questionnaire for sexual function has been the FSFI.

The cohort’s mean menarche of 12.7 years was found to be comparable to the mean of 13 years in the general Swedish population. The coital debut was substantially earlier in the present study compared to earlier studies by Euleteri et al. and Kyrklund et.al who showed a mean debut at 22.7 and 18 years of age, respectively [24, 25]. Swedish female teenagers have been shown to on average have their coital debut at age 16[26]. Two of the fourteen women (14.3%) had been pregnant, and no vaginal deliveries were reported. In 2013 Mantoo et al. suggested that birth rates in ARM female patients were equivalent to that of the general population [27]. Importantly, our cohort is relatively young, therein plausibly impacting the number of pregnancies and birth rate as well as sexual function outcomes in our study. In 2022, the mean age of first-time mothers in Sweden was found to be between 28.3 and 31.2 years, notably later than the women in our study population. In 70% of the women in our cohort, the impact of the ARM on their sexual life was limited.

Sexual function is multifactorial, and we show that at least low HRQoL is associated with impaired sexual function. These findings are in line with previous research by Kyrklund et. al who described a negative effect of low HRQoL on sexual function in females [11].

Interpreting HRQoL and sexual function of patients with congenital malformations is challenging as these patients were born with their condition and thereby their reference of HRQoL and sexual function could arguably differ from that of the reference population. In our previous research, from where we extrapolated the HRQoL data, the HRQoL results were gravely impaired compared to normative data. This diminished HRQoL can hypothetically explain our cohort’s impaired sexual function results where they scored lower than the controls in all domains. The “desire” domain was the domain in which our patients reported the lowest scores. Qualitative responses in our questionnaire such as “ashamed of my appearance” can speculatively factor into this poor outcome.

Composite outcomes in this setting have been scantily studied. Despite poor reported bowel function in this group, we could not find a correlation between it and sexual function. We did, however, find a correlation between the overall sexuality satisfaction question and the PFSF total score. This would suggest that a 1-question questionnaire could suffice in a clinical setting for general evaluation.

### Limitations

Small study populations and multi-variables make these analyses challenging. Selection bias of the responders was minimized as participants met the inclusion criteria before being asked to answer the questionnaires. However, it can be speculated that patients doing worse in their HRQoL or PFSF would be inclined to participate thus creating a selection bias. There was also a heterogenicity within our cohort mainly pertaining to the different ARM subtypes which we were unable to stratify for.

The 46.7% response rate was a limitation. This made it difficult to further stratify our cohort by ARM subtype. Several domains in the PFSF showed a trend of being significantly inferior to the controls and they might have been if the study population had been larger. Furthermore, the visual trend seen in BFS > 17 and PGWBI > 72 may have significantly impacted PFSF if the study population had been larger (Fig. 4). Furthermore, patients in our cohort were statistically younger than the reference control group which could explain, at least in part, their poorer PFSF score reports.

## Conclusion

Sexual function in adult females was comparable to healthy controls except for the “desire” item where the cohort reported significantly poorer outcomes. The cohort’s sexual function had a direct association with their reported HRQoL where individuals with worse HRQoL also had poorer sexual function. Coital debut for our cohort was at a younger age than previously described in other study populations and successful pregnancies were reported in this series. Further controlled studies with a focus on pregnancies, deliveries and their complications are deemed essential. Finally, larger studies are needed to evaluate sexual and reproductive function for a greater age span of adult women with a history of ARM.

## Supplementary Information

Below is the link to the electronic supplementary material.

Supplementary file1 (DOCX 14 KB)

## Data Availability

Artificial Intelligence (AI) and AI-assisted technologies were not used in the preparation of this manuscript. No datasets were generated or analysed during the current study.

## Abbreviations

ACE: Antegrade continence enema
ARM: Anorectal malformation
BFS: Bowel function score
FSFI: Female sexual function index
HRQoL: Health-related quality of life
IQR: Interquartile range
PFSF: Profile of female sexual function
PGWBI: Psychological general well-being index
PSARP: Posterior sagittal ano-recto-plasty
SD: Standard deviation
VACTERL: Vertebral-anal-cardiac-tracheo-esophageal-renal-limb
