# Supplementary material for: Sexual function and fertility in young female adults surgically treated for anorectal malformations
Source: Pediatr Surg Int. 2024 Oct 10;40(1):269. doi: 10.1007/s00383-024-05847-1 (PMC11467109; doi:10.1007/s00383-024-05847-1)
Supplement: Supplementary file 1 — Supplementary file1 (DOCX 14 KB) [file 383_2024_5847_MOESM1_ESM.docx]

**Supplementary Material:**

| **PFSF Domain** | **Cohort, Mean (SD)** | **Control Data, Mean (SD)** | **p-value** |
| --- | --- | --- | --- |
| PFSF Total Score | 56.7 (20.9) | 69.4 (15.9) | 0.061 |
| Self-Image | 45.8 (32.0) | 64.0 (20.3) | 0.077 |
| Responsiveness | 71.7 (16.6) | 78.2 (16.6) | 0.212 |
| Concern | 60.6 (32.2) | 73.9 (20.2) | 0.183 |
| Pleasure | 52.9 (29.3) | 68.5 (26.1) | 0.096 |
| Orgasm | 63.3 (27.2) | 66.4 (25.3) | 0.711 |
| Arousal | 64.4 (29.0) | 78.3 (21.0) | 0.131 |
| Desire | 38.0 (22.7) | 56.9 (19.5) | **0.015** |

**Supplementary table 1.** Summary data for cohort PFSF measures compared to the reference group. (Abbreviations: Profile of Feale Sexual Function, PFSF; Strandard Deviation, SD)
